# Human Neural Stem Cell Induced Functional Network Stabilization After Cortical Stroke: A Longitudinal Resting-State fMRI Study in Mice

**DOI:** 10.3389/fncel.2020.00086

**Published:** 2020-04-07

**Authors:** Anuka Minassian, Claudia Green, Michael Diedenhofen, Stefanie Vogel, Simon Hess, Maren Stoeber, Marina Dobrivojevic Radmilovic, Dirk Wiedermann, Peter Kloppenburg, Mathias Hoehn

**Affiliations:** ^1^In-Vivo-NMR Laboratory, Max Planck Institute for Metabolism Research, Cologne, Germany; ^2^Biocenter, Institute for Zoology, University of Cologne, Cologne, Germany; ^3^Cologne Excellence Cluster on Cellular Stress Responses in Aging-Associated Diseases (CECAD), University of Cologne, Cologne, Germany; ^4^Department of Histology and Embryology, School of Medicine, University of Zagreb, Zagreb, Croatia; ^5^Department of Radiology, Leiden University Medical Center, Leiden University, Leiden, Netherlands

**Keywords:** distal MCA occlusion, mouse, moderate severity stroke model, resting-state fMRI, human neural stem cells, functional connectivity, stroke-induced hyperconnectivity, neuronal differentiation

## Abstract

Most stroke studies dealing with functional deficits and assessing stem cell therapy produce extensive hemispheric damage and can be seen as a model for severe clinical strokes. However, mild strokes have a better prospect for functional recovery. Recently, anatomic and behavioral changes have been reported for distal occlusion of the middle cerebral artery (MCA), generating a well-circumscribed and small cortical lesion, which can thus be proposed as mild to moderate cortical stroke. Using this cortical stroke model of moderate severity in the nude mouse, we have studied the functional networks with resting-state functional magnetic resonance imaging (fMRI) for 12 weeks following stroke induction. Further, human neural stem cells (hNSCs) were implanted adjacent to the ischemic lesion, and the stable graft vitality was monitored with bioluminescence imaging (BLI). Differentiation of the grafted neural stem cells was analyzed by immunohistochemistry and by patch-clamp electrophysiology. Following stroke induction, we found a pronounced and continuously rising hypersynchronicity of the sensorimotor networks including both hemispheres, in contrast to the severe stroke filament model where profound reduction of the functional connectivity had been reported by us earlier. The vitality of grafted neural stem cells remained stable throughout the whole 12 weeks observation period. In the stem cell treated animals, functional connectivity did not show hypersynchronicity but was globally slightly reduced below baseline at 2 weeks post-stroke, normalizing thereafter completely. Our resting-state fMRI (rsfMRI) studies on cortical stroke reveal for the first time a hypersynchronicity of the functional brain networks. This *hyper*synchronicity appears as a hallmark of mild cortical strokes, in contrast to severe strokes with striatal involvement where exclusively *hypo*synchronicity has been reported. The effect of the stem cell graft was an early and persistent normalization of the functional sensorimotor networks across the whole brain. These novel functional results may help interpret future outcome investigations after stroke and demonstrate the highly promising potential of stem cell treatment for functional outcome improvement after stroke.

## Introduction

Stroke is one of the major causes of permanent disabilities and death within the Western world (Mackay and Mensah, 2013). The only clinically accepted therapy is the use of recombinant tissue plasminogen activator (rtPA) treatment for lysing the blood clots. This, however, is recommended only during the first hours of the acute stroke period, and due to serious risks for dangerous side effects, only a small proportion of patients profit from this rtPA lysis. Therefore, novel, additional therapeutic strategies are urgently needed.

The advancement of stem cell biology during the past 20 years has raised great expectations, and stem cell grafting after stroke has been investigated in many pre-clinical studies over the past years (Baccigaluppi et al., 2009; Lindvall and Kokaia, 2011; Minnerup et al., 2011). Here, reports on positive outcome improvements were mainly based on behavioral studies not explaining the mechanisms of treatment success (Oki et al., 2012; Tornero et al., 2013; Doeppner et al., 2017).

Reports on studies applying functional stimulus-based magnetic resonance imaging (fMRI) and resting-state fMRI (rsfMRI) have provided information on the dynamics of functional deficit (Dijkhuizen et al., 2001, 2003; Weber et al., 2008; van Meer et al., 2010a,b, 2012) and on the improvements after stem cell treatment (Ramos-Cabrer et al., 2010; Green et al., 2018). In these studies, it has been demonstrated that not only the focal ischemic tissue is functionally affected but that also far-range brain regions, even including the contralateral hemisphere, are affected by functional neuronal network alterations. Most of these studies, both on spontaneous stroke development and stem cell treatment of stroke, were performed using the filament occlusion model of the middle cerebral artery (MCAO) in rats and mice, a model that typically produces large ischemic lesions covering often almost the whole hemisphere. This MCA occlusion (MCAO) model of large lesions challenges the long-term survival and must be seen as a severe stroke type model (probably best reflecting the clinical category of high severity stroke).

As an alternative, distant occlusion of the MCA (dMCAO) by electrocoagulation has been proposed as a highly reproducible and robust stroke model with low mortality (Herrmann et al., 2005; Kuraoka et al., 2009), with good control of infarct volume, well suited for studies of neuro repair and stem-cell-mediated regeneration processes. In this dMCAO model, only a well-circumscribed cortical lesion of a small extent is produced allowing to categorize it in a mild to moderate stroke condition. As ischemic lesions of small size in the vast majority of human strokes are the primary target of recovery therapies, the dMCAO model presents a clear advantage over the large ischemic lesions of the MCAO model when it comes to translational perspectives. We have recently characterized the anatomical alterations of this moderate severity stroke model over 3 months, a time window relevant for regeneration studies (Minassian et al., 2019). The observed alterations mainly included cortical vasogenic edema in the acute/subacute phase followed by cortical tissue loss in the chronic phase, and shift of the hippocampus towards the freshly available tissue void.

In the present investigation, we aimed to study the functional aspects of this mild/moderate severity stroke model in mice by characterizing the spontaneous development of functional deficit. For this purpose, we assessed the most affected functional sensorimotor networks with rsfMRI and found a pronounced hypersynchronicity of the neuronal networks. Furthermore, we investigated the effect of stem cell grafting for the stabilization or recovery of the functional networks. Human neural stem cells (hNSCs) were implanted, and the therapeutic potential of stem cells to induce a widely stable and persistent normalization of the functional networks under these conditions of limited cortical damage is reported.

## Materials and Methods

### Animal Handling

A total of 53 homozygous male NMRI-Foxn1 mice with Tyr^c^ albino background were purchased from Janvier Labs (Le Genest-Saint-Isle, France) and housed under a 12:12 h light:darkness cycle with access to food and water *ad libitum*. In the first week of the longitudinal experiment, mice were 8 weeks old and weighed 30 ± 2 g. Animals were divided into four different experimental groups. A total of 32 mice underwent stroke induction using occlusion of the dMCAO by electrocoagulation. 18 mice were left untreated after stroke induction (“*untreated stroke group*”) and the other 14 dMCAO mice received human stem cells by stereotactic implantation 1 week after stroke induction (“*cell treated* *stroke group*”). For controls, 12 mice underwent sham occlusion of the distal MCA (“*sham stroke group*”), and further nine mice were implanted with hNSCs in the naïve brain (“*cell treated healthy group*”).

All animal experiments were conducted following the German Animal Welfare Act guidelines and approved by the local authorities from LANUV (Landesamt für Natur, Umwelt und Verbraucherschutz Nordrhein Westfalen). The animal permission was approved under license 84-02.04.2014.A370.

### Experimental Protocol

**Figure 1:**
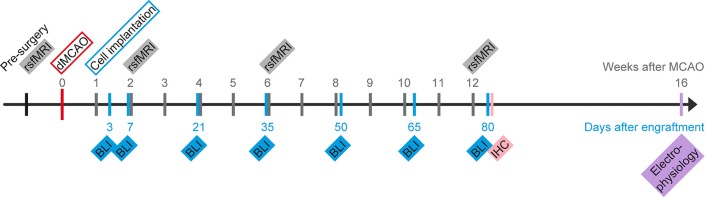
Schematic presentation of the project timeline. Time of stroke induction by distal occlusion of the middle cerebral artery (dMCAO) serves as day zero for the MRI scans. Human neural stem cells (hNSCs) were intracortically implanted 7 days later. Repetitive measurements of bioluminescence imaging (BLI) for graft vitality monitoring and resting-state fMRI (rsfMRI) for functional connectivity networks of the brain are indicated on a separate time ray with day 0 at the day of implantation. At 12 weeks after stroke induction, some of the mice were sacrificed to process brains for histology (IHC). Weeks after stroke and days after engraftment, respectively, are marked along the timeline.

Figure 1 depicts the time profile of the experimental protocol. All animals were scanned for anatomical MRI and rsfMRI before surgery, and at 2, 6 and 12 weeks after stroke (or sham) induction. Additionally, anatomical scans were performed at 48 h after stroke induction, serving as a demonstration of successful MCA occlusion and for demonstration of lesion size. For the group that included cell transplantations in the ischemic brain, cells were implanted 1 week after stroke induction. The group in which cells were implanted in the naïve brain, no stroke induction nor sham occlusion took place, therefore the MR images were acquired at 1, 5 and 11 weeks after cell implantation to match with the experimental timing of both ischemic groups. Bioluminescence imaging (BLI) measurements took place at 3, 7, 21, 35, 50, 65 and 80 days after cell implantation. All nine mice from the *cell treated* *healthy group* and seven mice from the *cell treated* *stroke group*, eight mice from the *untreated stroke group*, and five mice from the *sham stroke group* were perfused for immunohistochemistry at the end of the 12 weeks observation period. Of the remaining seven mice from the *cell treated stroke group*, five of them were used for patch-clamp electrophysiological recordings of the cell graft. The patch-clamp experiments were performed 4 weeks later at 16 weeks, as we suspected from earlier experiments following* in vivo* differentiation of the same stem cell line that mature neurons are showing up only after 3 months after implantation (Tennstaedt et al., 2015a). The remaining mice were sacrificed at the end of the observation period. Further, to assess the stability and reproducibility of the resting-state functional MRI, a separate group of 10 healthy mice were scanned three times, with a 10-day separation between scans.

### Stroke Induction

Anesthesia was induced in a knock-out box with an isoflurane concentration of 4% in an atmosphere of 70:30% N_2_O:O_2_. Mice were placed on a warm plate to control body temperature during surgery, isoflurane concentration was lowered to 2%, and the breathing rate was kept above 80 bpm at all times. Surgery for dMCAO followed an established protocol as previously described (Minassian et al., 2019). Analgesia was induced by Rimadyl (50 mg/ml Carprofen, Zoetis, Berlin, Germany) diluted 1:100 in saline solution and injected subcutaneously as a dose of 4 mg/kg body weight. A 5 mm incision was made between the right ear and eye, the temporal muscle was exposed and detached from the skull in its apical and dorsal ends, flapping it downwards to expose the bone. The skull was cleared from tissue and the distal end of the MCA was identified, rostral to the retro-orbital sinus. The bone was drilled thin above the intended coagulation site, and the last layer of bone was manually removed with thin forceps to expose the artery. The distal MCA was coagulated using an electrocoagulation forceps set to 7 W (bipolar) and checked for lack of reperfusion 30 s after coagulation. The temporal muscle was flapped back into its original position and the skin sutured with silk thread (RESORBA Medical, Nürnberg, Germany). Analgesia was maintained with Tramal (100 mg/ml Tramadol hydrochloride, Grünenthal, Stolberg, Germany) in drinking water (1 mg/ml) for the following 3 days.

For the sham surgery, the forceps tips were kept more separated from each other to avoid coagulation of the MCA, all other steps were followed as for the original surgery. No mortality was observed in any of the 44 mice after dMCAO or sham surgery, and all of them reached the end of the observation period of 12 weeks.

### Cell Culture, Cloning, and Transduction

HNSCs (Thermo Fisher Scientific, Waltham, MA, USA), originally derived from the NIH-approved human embryonic stem cell line H9 (Zhang et al., 2001) were used for implantation. Cells had been lentivirally transduced to express the two optical imaging reporters: firefly luciferase (Luc2) for *in vivo* BLI and enhanced green fluorescent protein (eGFP) for fluorescence imaging on tissue sections, as described earlier (Tennstaedt et al., 2015a).

The T2A peptide was used for self-cleaving. The construct was placed under the regulation of the constitutive promoter EF1α to express eGFP and Luc2 throughout the entire life of the cell. The final plasmid design for transduction was pCDH-EF1α-Luc2-T2A-eGFP (Tennstaedt et al., 2015a).

The H9 derived NSCs were maintained at 37°C and 5% CO_2_ according to the manufacturer’s protocol (Life Technologies, Carlsbad, CA, USA), and cells were cultured at a density of 5 × 10^4^ cells/cm^2^ on a Geltrex matrix (Thermo Fisher Scientific, Waltham, MA, USA) in StemPro NSC SFM complete medium, consisting of KnockOut DMEM/F12 medium, 2 mM GlutaMax, 20 ng/ml of basic fibroblast growth factor (bFGF) and epidermal growth factor (EFG), and 2% of StemPro Neural Supplement (all from Thermo Fisher Scientific, Waltham, MA, USA). The cells were passaged every 3 days using StemPro Accutase (Thermo Fisher Scientific, Waltham, MA, USA) to maintain them in an undifferentiated state.

### Cell Transplantations

Seven days after stroke induction, mice were anesthetized with isoflurane in 70:30% N_2_O:O_2_, and 4 mg/kg Carprofen was injected subcutaneously to induce analgesia. The animal was placed on a warming pad to keep its body temperature stable at 37°C and the temperature was controlled with a rectal probe. The head was fixed in a stereotactic frame (Stoelting, Dublin, Ireland). An incision in the skin was made to expose the skull, and Bregma was localized. A hole was drilled at the following coordinates relative to Bregma: AP +0.5; ML 1.8 (in the right hemisphere); DV 0.8 from the brain surface. The coordinates had been modified from Oki et al. (2012), to fit our settings of cortical implantation next to the ischemic lesion location.

The homogenous cell suspensions were kept on ice during surgery and were injected into the brain using a NanoFil syringe for microinjection (World Precision Instruments, Sarasota, FL, USA) with a 33G needle using a micropump system with an injection flow rate of 150 nl/min. A total volume of 1 μl containing 150,000 cells was injected and the needle was kept in place for 5 min after injection, to allow decantation of cells in the injection canal and to avoid cell withdrawal together with the retraction of the needle. The wound was closed with silk suture (RESORBA Medical) and the stitches were removed 3 days after surgery, before the first BLI measurement. Analgesia was maintained with Tramal (100 mg/ml Tramadol hydrochloride) in drinking water (1 mg/ml) for the following 3 days.

### Bioluminescence Imaging Acquisition and Analysis

Solutions of D-Luciferin (50 mg/ml) were prepared to dilute the powder contained in one vial of D-luciferin sodium salt purchased from Synchem (Felsberg, Germany) into 20 ml of Dulbecco’s phosphate-buffered saline (PBS, Life Technologies, Carlsbad, CA, USA). Solutions were filtered through a 0.2 μm syringe filter (VWR, Darmstadt, Germany) before use and stored at −20°C. Before *in vivo* measurements, aliquots were thawed and adapted to room temperature before injecting into the animal. Animals were injected intraperitoneally with a dose of 300 mg/kg body weight D-Luciferin. After injection, anesthesia was immediately induced with a mixture of 2% isoflurane in 70:30 N_2_O:O_2_ atmosphere. When no longer conscious, animals were immediately placed inside the Photon Imager IVIS SPECTRUM CT (Perkin Elmer, Waltham, MA, USA) under the same gas mixture conditions of anesthesia. The time lag between substrate injection and the beginning of image acquisition was kept constant at 3 min. The image acquisition protocol included one-photon emission measurement every 2 min, recorded for 30 min (Tennstaedt et al., 2015a).

*In vivo* BLI data was analyzed using the Living Image Software^®^ (Perkin Elmer, Waltham, MA, USA). In brief, the photon emission signal was color-coded according to magnitude, and overlaid to a black and white photographic image of the field of view containing the animal and a tritium standard, placed next to the animal, to control for signal variation in photon detection. The dynamics of photon emission was plotted to ensure that the maximum photon emission fell within the 30 min time window. When such a condition was not met, the animal’s measurement was repeated within the same week. The total flux was measured in photons per second (ph/s) at the moment of maximum emission within a circular region of interest (ROI) covering the whole head of the mouse. The size and location of the ROI was preserved in subsequent measurements for all animals.

### Immunohistochemistry

Mice were perfused transcardially under 4% isoflurane anesthesia with 20 ml PBS followed by 20 mL 4% phosphate-buffered paraformaldehyde (PFA) solution. The brain was carefully removed with a surgical spatula and incubated overnight in 4% PFA solution at 4°C. Subsequently, the PFA was replaced by a 30% sucrose solution, and the brain was incubated for 2 days at 4°C. On the third day, brains were shock-frozen in −40°C cold 2-methyl butane (Carl Roth, Karlsruhe, Germany) and stored at −80°C. Sections of 30 μm were cut in the coronal plane with a cryostat (Leica Biosystems, Wetzlar, Germany), mounted on Superfrost glass slides (Thermo Fisher Scientific, Waltham, MA, USA) and stored at −20°C.

Tissue sections were washed in PBS, and antigen retrieval was conducted by incubation in −20°C acetone for 20 min (for GFAP detection) or in 80°C sodium citrate (ph 6.0) for 30 min (for HuNu or NeuN detection). Next, sections were washed with PBS and incubated in blocking solutions containing PBS, 0.25% Triton X-100 (Carl Roth) and 5% normal donkey serum (Jackson ImmunoResearch, West Grove, PA, USA) for 1 h at room temperature. The primary antibodies were diluted in blocking solution and incubated overnight at 4°C in a wet chamber. The following primary antibodies were used: mouse anti-GFAP (1:200; Sigma–Aldrich, St. Louis, MO, USA), mouse anti-HuNu (1:100; Millipore, Burlington, MA, USA), chicken anti-NeuN (1:100; Synaptic System) and rabbit anti-GFP (1:200; Invitrogen, Carlsbad, CA, USA). The secondary antibodies were diluted together with the nuclear stain Hoechst 33,342 (1:1,000; Sigma-Aldrich, St. Louis, MO, USA) in blocking solution and incubated in a wet chamber at room temperature for 2 h. The following secondary antibodies were used: Cy5 donkey anti-mouse (1:200; Jackson ImmunoResearch, West Grove, PA, USA), Cy5 donkey anti-mouse (1:100; Jackson ImmunoResearch, West Grove, PA, USA), Cy3 donkey anti-chicken (1:100; Jackson ImmunoResearch, West Grove, PA, USA) and Alexa Fluor 488 donkey anti-rabbit (1:200; Thermo Fisher Scientific, Waltham, MA, USA). Finally, tissue sections were washed with PBS followed by distilled water and mounted with Cytoseal XYL (Thermo Fisher Scientific, Waltham, MA, USA).

### Electrophysiological Measurements

Whole-cell patch-clamp recordings were essentially performed as described previously (Tennstaedt et al., 2015a; Vogel et al., 2019). Details are provided in the Supplementary File 1.

### MRI Data Acquisition

MRI measurements were performed with a 9.4 Tesla animal scanner (Bruker Biospin, Ettlingen, Germany) with a 20 cm horizontal bore diameter, equipped with actively shielded gradient coils. RF transmitting and signal receiving was performed with a helium-cooled mouse ^1^H quadrature cryogenic surface coil (CryoProbe, Bruker Biospin). Paravision 6.01 (Bruker Biospin) was used to execute the MRI acquisition protocols. The animal was placed in an animal holder equipped with a mask supplying the breathing gases. The mouse head was fixed with teeth and ear bars. The animal’s body temperature was measured *via* a fiber optic rectal probe (1025T System, SA Instruments, Stony Brook, NY, USA) and kept constant at 37 ± 1.0°C by an adjustable water circulating system (medres, Cologne, Germany). Additionally, the breathing rate was continuously monitored (1025T System), recorded (DASYlab Software, Measurement Computing, Norton, USA), and synchronized with the MR image recording.

#### Anatomical Brain Imaging

A 3-plane pilot reference scan using a fast low angle shot (FLASH) was acquired to confirm the correct positioning of the mouse head, followed by a FieldMap with consecutive local shim to optimize magnetic field homogeneity and image quality. Next, a high-resolution anatomical T2-weighted turboRARE (turbo-rapid acquisition with relaxation enhancement) data set was acquired with a RARE factor of 8 and two averages. The field-of-view (FOV) was set to 17.5 × 17.5 mm^2^ and 48 coronal slices of 0.3 mm thickness without inter-slice gap were placed to cover the whole mouse brain. The matrix dimension was set to 256 × 256, repetition time (TR) to 5,500 ms and echo spacing to 10.8 ms, resulting in an effective echo time (TE) of 32.5 ms. The in-plane resolution was (68 μm)^2^ and a bandwidth of 39.062 Hz. A multi-slice multi-echo (MSME) spin-echo sequence was recorded for quantitative T2 evaluation and visualization of hyperintense signals in the brain. MSME acquisition parameters were as follows: TE = 11 ms, TR = 3,000 ms, number of echoes = 16, the scan time of 6 min 24 s, number of averages was 1, matrix size of 128 × 128, FOV of 18 × 18 mm, the in-plane spatial resolution of 140 (μm)^2^, the bandwidth of 50,000 Hz, slice thickness of 0.5 mm, number of slices was 11, coronal orientation with an inter-slice gap of 0.5 mm.

#### Resting-State fMRI

A bolus of 0.1 mg/kg body weight Medetomidine (Domitor^®^, Pfizer), suspended in 250 μl saline solution, was administered subcutaneously 15–20 min before the functional imaging scan, with subsequent reduction of isoflurane level to 0.5%, following an earlier reported protocol (Green et al., 2018, 2019). A gradient-echo echo-planar imaging (GE-EPI) sequence was used for rsfMRI with the following parameters: FOV: 17.5 × 17.5 mm^2^, matrix size: 96 × 96, in-plane resolution: 182 × 182 μm^2^, TR = 2,840 ms, and TE = 18 ms. One-hundred and five image sets were acquired with 16 slices each, with a slice thickness of 0.5 mm and an inter-slice gap of 0.1 mm, recorded non-interleaved and covering the whole forebrain, starting only after a minimal time of 10 min on reduced isoflurane levels.

### MRI Data Processing

Python 2.7.6, Matlab R2014b, and Linux Ubuntu 16.04 LTS shell scripts were used for programming. FMRIB Software Library (FSL 5.0.9), Advanced Normalization Tools (ANTS 2.1.0), and ImageJ 1.48 s with macros (scripting language) and plugins (Java 1.6) were used to process the MRI data. All images acquired with Paravision 6.01 software were first converted to NIfTI format and scaled up by a factor of 10 in each dimension to be able to be processed in software developed for human brain studies, e.g., FSL BET. Each rsfMRI dataset was temporally averaged, and the resulting mean image was brain extracted with FSL BET. The mask resulting from the brain extraction was applied to all volumes of the rsfMRI dataset, which were slice-wise motion-corrected with custom-made shell scripts employing FSL MCFLIRT. The transformation parameters were saved as text files (.par) and used later in the analysis of regressors. Regressors for breathing, motion correction, and drift variations (of 1st, 2nd and 3rd order) were generated from the physiological monitoring data (see above), the output files from MCFLIRT (.par files) and drift functions, respectively. The functional data fluctuations (correlating with respiration, motion correction, and drifting) were regressed out slice-wise using FSL_Regfilt. Subsequently, datasets were smoothed in-plane with a Gaussian filter of FWHM = 0.3 mm using FSL’s SUSAN to enhance the spatial signal-to-noise ratio, and bandpass filtered between 0.01 and 0.08 Hz (Biswal et al., 1996).

For the data analysis, major regions of interest were chosen that are known to be most affected by the dMCAO, together with other ROIs that play an important role in brain function due to their association with the affected cortical areas. The following five regions on each hemisphere were determined for pairwise correlation: the motor cortex (M), including M1 and M2; the primary somatosensory cortex (SS1); the secondary somatosensory cortex (SS2); the thalamus (Th), and the caudate-putamen (CPu). These regions of interest were extracted from a publicly available atlas, the Australian Mouse Brain Mapping Consortium atlas (AMBMC).

Next, the AMBMC selected labels were aligned to the functional data. For that purpose, the anatomical dataset was first converted to NIfTI format (scaled up 10×), brain extracted with FSL’s BET, and bias-corrected with FSL’s FAST. It was then coregistered to an in-house generated structural nude mouse brain template (*n* = 21) using an affine registration with 12 degrees of freedom for the pre-surgery images. This template had been previously coregistered to the AMBMC atlas. For the time points at 2, 6 and 12 weeks post-stroke induction, a different strategy was employed due to the earlier observed continuous substantial shifts of the hippocampus during the 12 weeks after dMCAO (Minassian et al., 2019): the hippocampus was manually delineated and the results applied in a point set expectation nonlinear registration which was the input of a subsequent diffeomorphic nonlinear registration. The resulting matrix of the affine transformation (FSL FLIRT) or the warp-field of the combined affine and diffeomorphic transformation (ANTs), were combined with the atlas-to-template transformation matrix and applied to the selected ROIs represented as labels in the AMBMC. Such labels were then transformed and aligned with the functional data.

Finally, the ROIs extracted from the atlas for both hemispheres were merged in a hyper stack. The datasets were used to calculate group analysis on the functional connectivity strength within each ROI and among ROIs, the latter for an assessment of the correlation between different brain regions, using FSLNets. The mean time course of all voxels within an ROI was calculated for each animal and stored in a text file. Group-wise full Pearson correlation between pairs of ROIs of the average time series was calculated with FSLNets (V0.6) and resulting values are displayed as z-scores in matrix form.

Lesion volume calculation: the hyperintensity in the cortical lesion was manually outlined on each third T2-weighted coronal section. Lesion sizes on the individual sections were added up and multiplied by the interslice distance. This was performed for each animal in each group at all measured time points, i.e., at 48 h after stroke, at 4, 6, and 12 weeks after stroke.

### Statistics

For BLI measurements and T2-weighted MRI lesion volumes, ANOVA for repeated measurements was performed (SPSS Statistics version 22, IBM Corp., USA), after checking sphericity in the distribution. After controlling for statistically significant differences within averages of the same group, a *post hoc* Bonferroni test was applied to the different groups at all time points to check for differences between cohorts. For the functional connectivity matrices, the correlation between pairs of ROIs was calculated using a full Pearson correlation. Bootstrapping analysis to determine the 95% confidence interval was performed for the scatterplot data of the matrix elements of different time points. A *p*-value < 0.05 was considered significant. Data are presented as mean plus/minus standard deviation (SD).

## Results

### Lesion Characterization

**Figure 2:**
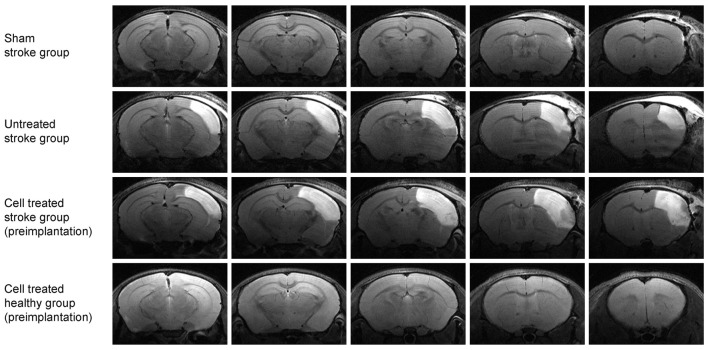
Multi-slice coronal T2-weighted MRI visualizing the acute cortical lesion. Multislice MR imaging shows the expansion of the lesion across the whole brain for representative animals of each of the four groups. The hyperintensity on the right hemisphere of the *untreated stroke group* (2nd row) and the *cell treated stroke group* (3rd row) depicts the ischemic territory at 48 h post distal MCA occlusion, showing a demarcated region between the brain surface and the corpus callosum. There is no clear difference in the lesion extent between these two-stroke groups (with and without cell engraftment). The *sham-treated stroke group* (1st row) is free of such extended cortical lesion except for a small hyperintense spot rather rostrally at the cortical surface at the site of the skull preparation. The fourth group of healthy animals with intracortically implanted human stem cells shows no anatomical alterations. The needle canal of the implantation site is barely visible on the second most rostral slice on the right hemisphere.

Figure 2 shows coronal brain sections for a representative animal for each of the four groups at 48 h after surgery by T2-weighted MRI. Sham occlusion of the MCA showed no hyperintensity due to vasogenic edema as no ischemia was produced (Figure 2, top row; *sham stroke group*). Only small hyperintense areas were observed in the cortical tissue adjacent to the skull in the *sham stroke group*, showing the accumulation of liquid as a result of the sham surgery. Vasogenic edema after ischemia, reflecting the lesion extent, was observed in all animals of the two groups undergoing distal MCA occlusion (Figure 2, second row, *untreated stroke group*; and third row, *cell treated stroke group*). Five T2-weighted MR images covering the rostrocaudal axis from Bregma coordinates AP −2.8 to AP +1.2 show the large cortical extent of the lesion for both dMCAO groups (Figure 2, rows 2 and 3), whereas no edema due to ischemia was observed in the sham occluded group (Figure 2, row 1). The fourth group of mice (*cell treated healthy group*) was also assessed by T2-weighted MRI (Figure 2, row 4) and served as a healthy control group at the 48-h time point since no invasive procedure had been applied to the brain at that point yet.

**Figure 3:**
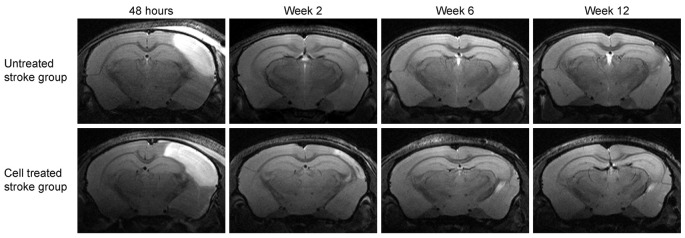
Lesion development on T2-weighted MRI at the level of the hippocampus. Representative animals for both groups involving stroke are shown. The image slice position at the level of the hippocampus was chosen equivalent to the second most caudal section in Figure 2. While the affected cortical area is visible at the acute time point at 48 h after stroke induction, the hyperintensity decreases with resolving vasogenic edema at 2 weeks. At 6 weeks and 12 weeks, both, the cortical tissue loss and the shift of the hippocampus towards the brain surface, become prominent.

Figure 3 shows the development of the lesion for the *untreated stroke group* and *cell treated stroke group* at all four-time points after the ischemic event, namely at 48 h, 2 weeks, 6 weeks and 12 weeks post distal MCA occlusion. Both groups present a closely similar development of the lesion in the chronic phase, as previously reported by us (Minassian et al., 2019). Note that, despite the *cell treated stroke group* undergoing an additional cell implantation surgery 7 days after dMCAO, the expression and resolution of the vasogenic edema is not different from the *untreated stroke group*. Quantification of the lesion reflecting hyperintensity on the T2-weighted MR images is listed in the Supplementary Table S1 showing closely similar lesion values for both groups with no statistically significant difference at all time points. In both groups, the thinned cortex becomes distinguishable after 2 weeks, progressing further until the 12th week. The hippocampal swelling and radial movement towards the skull is very evident at the end of the observation period. In contrast, the *sham stroke group* and the *cell treated healthy group* demonstrated neither T2-weighted hyperintensity nor anatomic reorganization such as hippocampal swelling or shifting (Supplementary Figure S1). The cell implantation did not result in visible marks on the MR images.

### Functional Connectivity

To assess the stability and reproducibility of the resting-state functional MRI, a separate group of 10 healthy mice was scanned three times, with a 10-day separation between scans. The results, analyzing the sensorimotor networks, are expressed as correlation factors among all the selected regions of interest. The correlation factors were z-scored before group average and displayed in numerically symmetric matrices. As depicted in Supplementary Figure S2, no relevant, major changes in connectivity across the whole sensorimotor networks could be observed between the three respective time points of the reproducibility experiment within the cohort, thus demonstrating the robustness of the rsfMRI experiment and data analysis throughout time as well as the stability of the group connectivity networks.

**Figure 4:**
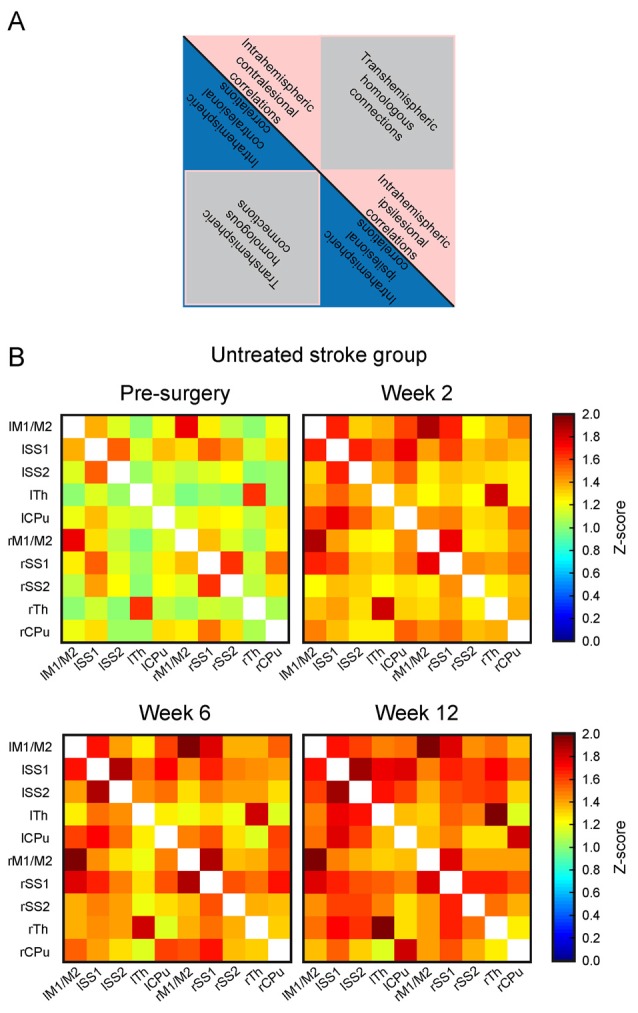
Functional connectivity matrices of the *untreated stroke group* at different time points. **(A)** Schematic of the matrix for general content interpretation: in such matrices, all lines along the vertical axis, and, equivalently, all columns along the horizontal axis describe all selected anatomical Region of interests (ROIs). As a result, each cell in the matrix contains the connectivity strength (expressed as *z*-score value) between the respective ROIs forming this cell. Due to symmetry of the correlations **(A)** vs. **(B)** is equivalent to **(B)** vs. **(A)** both triangles along the main matrix diagonal (indicated as a black line) contain identical information. The intrahemispheric correlations of the left (contralesional) hemisphere are located in the triangle of the upper left quadrant (blue or pink), the trans-hemispheric correlations are in the lower left quadrant (gray). The intrahemispheric correlations of the right (ischemic) hemisphere are depicted in the lower right quadrant. **(B)** Presentation of the functional connectivity matrices over time of the *untreated stroke group*. The matrix at the first post-stroke time point at 2 weeks shows strongly increased z-score values. During the following weeks, a further increase of the functional connectivity strength is noted. Note that the changes affect both hemispheres in the same way. Here, *lM1/lM2* stands for left primary and secondary motor cortices, *lSS1* stands for left primary somatosensory cortex (SS1), *lSS2* stands for left secondary somatosensory cortex (SS2), lTh for left Thalamus, *lCPu* for left Caudate Putamen; and similar for the right hemisphere regions: rM1/M2 stands for right primary and secondary motor cortices, *rSS1* stands for right SS1, *rSS2* stands for right SS2, rTh for right Thalamus, and* rCPu* for right Caudate Putamen. The horizontal axis describes the same selected ROIs in the aforementioned order.

**Figure 5:**
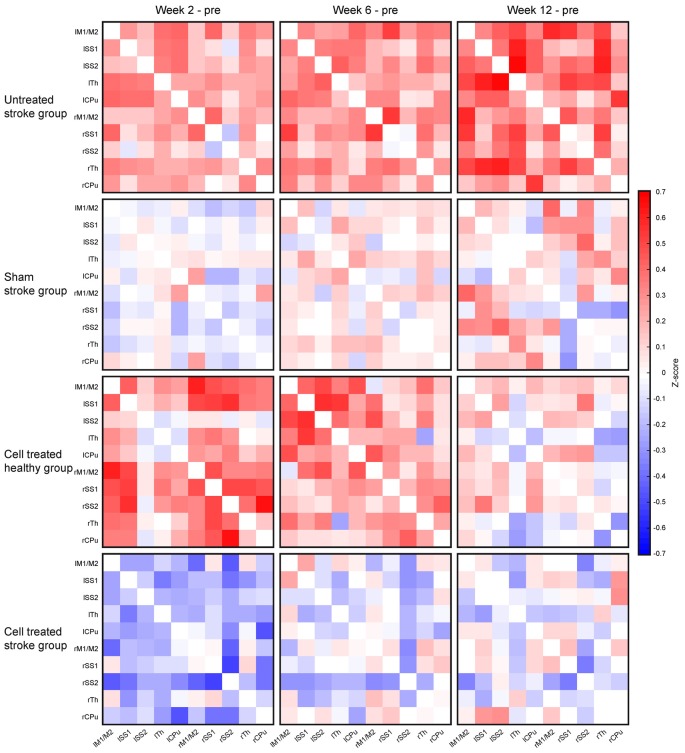
Changes of the functional connectivity matrices for all four experimental groups. The functional connectivity matrix obtained at the pre-stroke control time point was subtracted cell-wise from the matrices at the different individual time points after stroke induction, i.e., at 2 weeks, 6 weeks, and 12 weeks. The top row shows the continuous and strong increase of the functional connectivity for the *untreated stroke group*. The second row reflects the situation for the *sham-treated stroke group* with only small z-score fluctuations, showing difference values close to zero. In the third row, the situation for the *cell treated healthy group* is given, demonstrating a severe increase of z-score values above the pre-implantation control at 2 weeks, followed by a decrease at 6 weeks and approximation towards the baseline again at 12 weeks. Finally, the bottom row presents the difference matrices of the *cell treated stroke group*. This is the only group with a substantial reduction of the z-score values at 2 weeks, followed by a close approximation back to the baseline values again, as reflected by the faint coloring.

The matrices of the *untreated stroke group* over the 12 weeks are depicted in Figure 4. Before the surgical intervention, the global connectivity strength is closely similar to that of the *sham-treated stroke group* at the age of 2 months. In our earlier report on rsfMRI on transient MCAO (Green et al., 2018), we had defined a threshold to recognize a reliable change for a *z*-score value >0.2. This threshold equals 15% above the global value of the pre-stroke matrix for the *untreated stroke group*. At the first observation time point, 2 weeks after induction of cortical ischemic lesion, major increases of the sensorimotor network connectivities were present in the *untreated stroke group*, with an increase of the *z-score* values by 24% over baseline. This increase continued to 28% at 6 weeks and finally to 35% at 12 weeks. This increase is not limited to the ischemic hemisphere but covers rather equally the whole networks across both hemispheres. To better express the quantitative change over time, we present the difference matrices subtracting the values at the pre-stroke control time point from the individual post-stroke time points in Figure 5. The steady global z-score increase over time of the *untreated stroke group* is seen in Figure 5, top row.

The difference matrices for the *sham stroke group* are depicted in Figure 5, second row. There is only a slight, but not significant increase of *z*-score values over a period during the 12 weeks observation after sham MCA occlusion, confirming stable results of sham-operated animals, comparable to the strong reproducibility in healthy animals (see Supplementary Figure S2), over even longer periods.

Interestingly, implantation of neural stem cells into the healthy cortex in the *cell treated healthy group* led to a strong global connectivity strength increase at 2 and 6 weeks, well comparable to the increase of the stroke group, while renormalizing at the end of the 12 weeks observation. This is visualized with the different matrices in Figure 5, third row. When neural stem cells were implanted adjacent to the ischemic cortex at 1 week after distal MCA occlusion, no increase of the connectivity strength was seen in the *cell treated stroke group*, opposed to the situation of the *untreated stroke group*. Instead, the connectivity strength of the *cell treated stroke group* was globally slightly reduced at 2 weeks post distal MCA occlusion (Figure 5, bottom row). At 6 weeks, the global connectivity strength had re-increased approximating the baseline, pre-stroke values, which were almost completely reached at 12 weeks.

### Regression Analysis of Time Profile of Functional Connectivity Matrices

**Figure 6:**
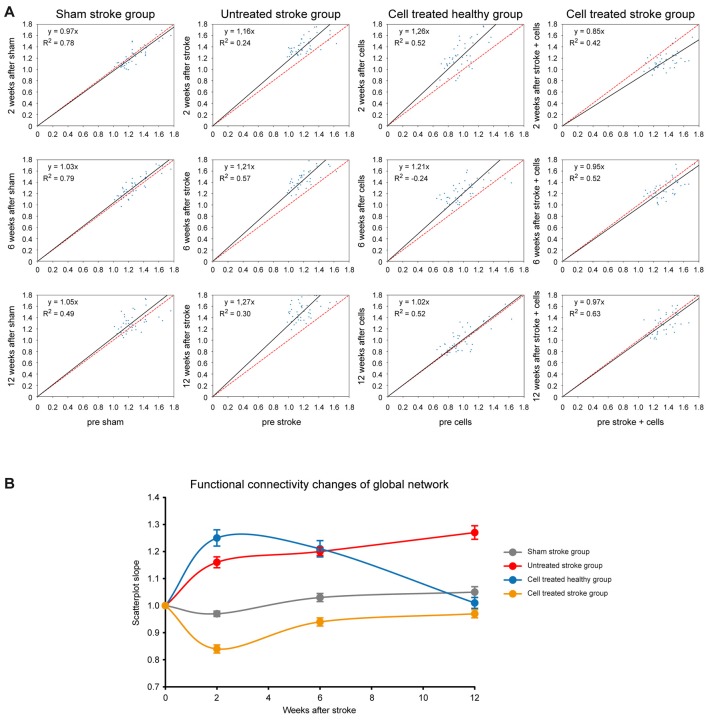
Changes of global network strength relative to baseline. **(A)** For all cells of the matrices, the z-score values were plotted on the horizontal axis for the baseline condition and the vertical axis for either the 2 weeks, or 6 weeks, or 12-week time point. The deviation of the fit from the identity line (given in red) indicates the increase or decrease in global functional network strength. The four columns depict the situations of the four different animal groups for the three time points. In the left upper corner of each plot, the linear equation with the slope of the linear fitting and the coefficient of determination (*R*^2^) are presented. **(B)** The slope of the fits in **(A)** was plotted against the respective time point for all four groups, depicting the strong connectivity increase of the *untreated stroke group* and the *cell treated healthy group*. The *sham-treated stroke group* presents no clear change at all, and the *cell treated stroke group* is characterized by a first strong decrease, followed by a recovery to the baseline condition. Error bars represent the 95% confidence interval from bootstrapping.

Rather than highlighting individual changes of correlations between specific ROIs in the matrices mentioned above, we aimed to quantitatively assess the overall functional differences after treatment. Scatter plots of all functional connectivity matrix elements between the pre-intervention time point (baseline) and the 2 weeks, 6 weeks, and 12-weeks time point, respectively, were generated. In the case of no changes of the individual matrix elements between two time-points, all points would be expected to lie on the central diagonal through zero with a slope of 1 (identity line). Deviation of the fitted slope from the central diagonal (slope = 1.0, the red line in Figure 6) hereby indicates the overall increase or decrease of the z-score values from the baseline to the later (post-intervention) time point for all four groups (Figure 6A). For the *sham stroke group* (first column in Figure 6A) no clear deviation from the identity line is observed for all three combinations, confirming the lack of interference of the sham surgery on the functional networks. In the *untreated stroke group* (second column in Figure 6A) a substantial increase from baseline is noted already at 2 weeks post-stroke induction, indicated by the slope of 1.16. This deviation continuously increased to 1.21 (6-week time point) and to 1.27 (12-week time point). The deviation from the identity line for the *cell treated healthy group* (Figure 6A, third column) is similar to the *untreated stroke group*, showing a slope of 1.26 at 2 weeks, but then continuously decreasing to 1.21 (6-week time point) and reaching the baseline again at 12 weeks (with a slope of 1.02).

Interestingly, the situation for the *cell treated stroke group* (Figure 6A, fourth column) is quite different as it does not reflect an addition of the network connectivity changes of both individual surgical procedures alone, i.e., stroke induction and cell implantation. Instead, there is a strong early decrease of the slope to 0.85 at 2 weeks post-stroke induction (and at 1-week post cell implantation), indicating a strong reduction of the functional network strength at this time. This decrease, however, well recovered to the baseline network strength at 6 and 12 weeks, as reflected by the slope normalization (0.95 at 6 weeks; 0.97 at 12 weeks).

For visualization purposes, slopes for all groups at all time points were translated into a graph where the network strength alterations can be easily compared (Figure 6B) while bootstrap analysis provided the corresponding 95% confidence intervals. As described above, the global connectivity strength of the *sham stroke group* remains stable. In contrast, the overall z-scores of the *untreated stroke group* show a steep increase at week 2 and values continue to rise steadily until week 12. The slopes of the *cell treated healthy group* rise even beyond values of the *untreated stroke group* at week 2, remain elevated at week 6 and turn back to baseline at week 12, as opposed to the continuous increase showed by the ischemic group. Finally, the *cell treated stroke group* shows only an early decrease of z-scores with recovery to baseline by the end of the observation period.

### Vitality of the Stem Cell Graft

**Figure 7:**
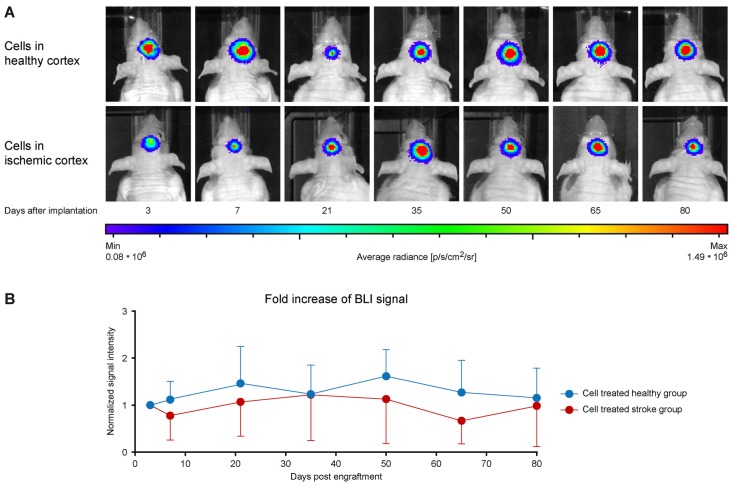
Stem cell vitality over time. **(A)** The bioluminescence images of representative animals from the healthy mice with stem cell implantation (upper row) and from the animals with stem cell implantation 7 days after stroke (lower row) show in both cases rather stable signal intensities at the seven measurements over the 12 weeks observation. **(B)** Quantitative analysis of the BLI signal intensities within both groups. BLI intensities were normalized to the first measurement time point after cell engraftment for each animal individually. Data are presented as mean ± standard deviation (SD). No significant changes are observed between both groups and over time. The stable BLI signal intensities demonstrate persistent graft viability during the whole 12 week observation period.

The hNSCs had been stably transduced to express luciferase under constitutive conditions. As the luciferase reaction is dependent on the presence of Adenosine triphosphate (ATP) and oxygen, the bioluminescence reaction is used to monitor cell viability over time. Seven days after stroke induction for the *cell treated stroke group* or 7 days after the baseline functional connectivity scans for the *cell treated healthy group*, 150,000 hNSCs were implanted into the primary motor cortex, adjacent to the edematous SS1, visualized as a hyperintense region on the T2-weighted MRI. BLI was performed 3 and 7 days after transplantation, and then every 2 weeks until the end of the 12 weeks observation period. At the first time point of BLI recording, the signal was strong for both groups and remained at this stable level with only little non-significant fluctuations throughout the 12 weeks observation, as depicted in Figure 7A for a representative animal from each group. The quantitative analysis of the BLI signal intensity is given in Figure 7B, where the similarity of signal intensity in both groups can be observed. The stability of the BLI signal over time demonstrates the stable graft viability in both, naïve and ischemic brains, without significant vitality loss.

### Characterization of Lineage Differentiation of the Graft

**Figure 8:**
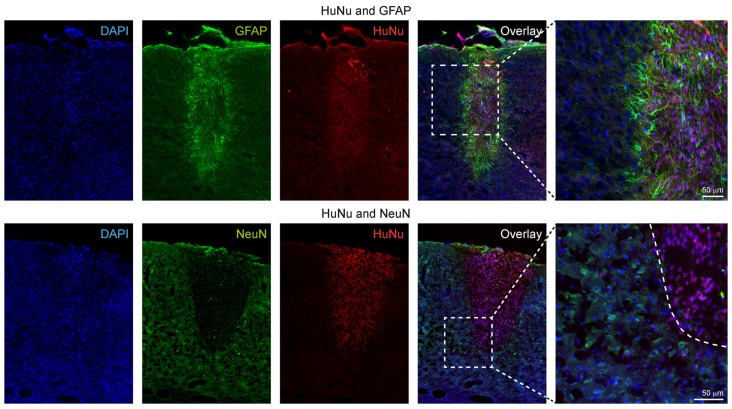
Immunohistochemical characterization of the human stem cell graft at 12 weeks after stroke induction. The upper row shows that all GFAP^+^ cells are negative for HuNu, depicting only the glial scar around the graft and indicating that the graft did not differentiate into the astroglial lineage. In the lower row, double staining of HuNu for the engrafted cells and NeuN for mature neurons was negative, indicating that most stem cells differentiating into the neuronal lineage (see further electrophysiological data on Figure 9) had not yet reached differentiation state of fully mature neurons at 12 weeks.

First, we characterized the glial reaction to the hNSC grafts. The astroglial scar at week 12 was detected by glial fibrillary acidic protein (GFAP) antibody staining, and detection of the engrafted cells in the brain sections was achieved by human nuclei (HuNu) antibody staining. GFAP^+^ reactive astrocytes were found both in the surroundings and in the core of the graft, forming a glial scar as shown in Figure 8, upper row. No co-localization of GFAP^+^ and HuNu^+^ cells was detected, excluding the presence of engrafted cells with astrocytic cell type. The astrocytic processes observed in the core of the graft are projections from the surrounding host reactive astrocytes or host migrating astrocytes.

Next, we used antibodies against the neuronal nuclei (NeuN) antigen which is exclusively expressed by mature neuronal cells to assess the differentiation stage of the engrafted hNSCs into the neuronal lineage. As opposed to our earlier study implanting the same cells into mouse brain after transient MCA occlusion using the filament occlusion technique, where we identified single cells at the border zone of the graft presenting a double stain, in the ischemic brains of the present study we did not observe double-positive cells for both HuNu and NeuN antibodies at 12 weeks. However, many host cells surrounding the graft presented a NeuN phenotype, indicating the constant presence of mature neurons in the host tissue, particularly next to the graft location (Figure 8, lower rows).

### Electrophysiological Graft Characterization

**Figure 9:**
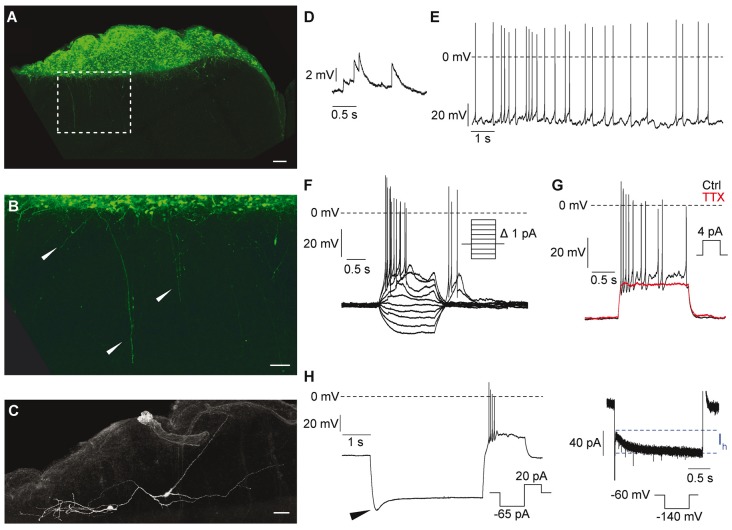
Electrophysiological and morphological properties of engrafted hNSCs recorded at 16 weeks after stroke induction and 15 weeks after grafting. **(A)** Enhanced green fluorescent protein (eGFP)-staining of the implanted graft. Scale bar: 100 μm. **(B)** Higher magnification of the dashed rectangle in **(A)** showing projections from the graft into the cortex. Scale bar: 50 μm. **(C)** Biocytin-labeling of recorded cells. Scale bar: 50 μm. **(D)** Excitatory postsynaptic potentials (EPSP) indicating functional synaptic connection. **(E,F)** Some cells generated spontaneous action potentials **(E)**, while others generated trains of action potentials only during depolarizing current injections **(F)**. **(G)** Blocking of action potentials by TTX (1 μM) indicates the expression of voltage-gated Na^+^ channels. Black trace: control; red trace: TTX. **(H)** Left: hyperpolarization of the cell to ~−140 mV induced a sag-potential (arrowhead) which is mediated by *I*_h_. Right: *I*_h_ elicited by a hyperpolarizing voltage-step to −140 mV.

To assess the functional state of hNSCs grafts, we characterized the electrophysiological properties of engrafted hNSCs-eGFP using whole-cell patch-clamp recordings in acute brain slices of mice at 16 weeks after the beginning of the observation period. Engrafted cells could be identified by their eGFP fluorescence (Figure 9A). More importantly, projections from the graft into the cortex could be observed (Figure 9B). For patch-clamp recordings, clearly recognizable and distinctly defined cell bodies were selected. During the recording, the cells were labeled *via* the patch pipette with biocytin-streptavidin for post-recording morphological examination. All cells which were labeled had a complex “neuron-like” morphology (Figure 9C). Accordingly, excitatory postsynaptic potentials (EPSP) could be detected indicating functional synaptic connections (Figure 9D). All of the recorded hNSCs had the ability to generate action potentials. Approximately 67% (8/12) of these cells generated action potentials spontaneously (Figure 9E), while the other 33% (4/12) of the cells generated trains of action potentials only during depolarizing current injections (Figures 9F,G). Typically, the action potentials were over-shooting and were blocked by TTX, showing that they were Na^+^-mediated (Figure 9G). While all recorded cells were spiking, current injection protocols revealed that neurons had developed different intrinsic electrophysiological properties such as the presence of a sag potential at hyperpolarized membrane potentials (Figure 9G, left) which is mediated by the h-current (I_h_, Figure 9H, right).

## Discussion

We have applied the distal MCA occlusion model to produce well-circumscribed cortical ischemic lesions of the same size, and implanted hNSCs adjacent to the lesion territory. In this cortical stroke model, the functional network changes after stroke have been characterized and a profound influence on the network stabilization by the grafted stem cells is reported. Stable graft vitality was successfully monitored and neuronal differentiation of the graft was demonstrated by electrophysiological characterization. This is the first report to the best of our knowledge of functional network changes in this cortical stroke model and, in particular, of stabilizing effects on the functional networks by implanted stem cell grafts.

### Functional Network Changes After Cortical Stroke

At the first observation time point, 2 weeks after induction of cortical ischemic lesion, substantial increases of the sensorimotor network connectivities were present in the *untreated stroke group*, with an increase of the z-score values by 24% over baseline, and continuing to reach even 35% at 12 weeks. Interestingly, this increase in functional connectivity strength was found globally, i.e., with no clear difference between both, ipsilateral, ischemic and contralateral, healthy hemisphere. The observation that both hemispheres appear to be equally affected by the focal lesion is in agreement with earlier studies on large stroke models using the filament occlusion model in the same strain of mice (Green et al., 2018). But in the present investigation, a strong and persistent connectivity increase was noted after stroke in contrast to earlier studies in mice (Green et al., 2018) and rats (van Meer et al., 2010b, 2012) which had reported a severe decrease in functional connectivity strength, also persisting over several weeks. This new finding of hyperconnectivity after stroke in the present investigation is not explained by differences in species or strains as Green et al. (2018) used the same strain of nude mice as in the present study. Data recording and analysis also followed the same protocols. However, the striking difference to earlier reports by van Meer et al. (2010b) on rats and by Green et al. (2018) on mice is the stroke model. While in those earlier studies, the filament MCAO was applied (both in rats and mice) leading to extensive, large ischemic territories covering often a major fraction of the ipsilateral hemisphere and encompassing both cortical and striatal territories, the presently used distal occlusion of the MCA resulted only in small, circumscribed cortical lesions with high reproducibility (Minassian et al., 2019). It thus appears likely to assign the difference in functional connectivity strength alterations to the severity of stroke damage, volume, and location of the lesion. It is noteworthy as an alternative explanation that not so much the lesion severity but the involvement of different brain regions could determine the development towards *hypo*synchronicity or *hyper*synchronicity. In all former reports of a connectivity decrease (van Meer et al., 2010a,b, 2012; Green et al., 2018, 2019), the ischemic territory encompassed the striatum, with or without cortical inclusion, whereas in the present study the connectivity increase is caused by a purely cortical lesion without any subcortical involvement. Thus, it will be of great interest to investigate in future studies the role of the striatum for the determination of whether functional networks show a *hypo*- or *hyper*synchronicity following stroke.

At any rate, the large hemispheric lesions, as produced by the filament occlusion model, result in a total brain-wide breakdown of the normal functional network condition while the brain responds to the smaller and milder lesion of the here used small, circumscribed cortical ischemia with an increased synchronization reflected by the strong hyperconnectivity. Such hyperconnectivity has also been reported in a rat trauma model of moderate severity, which may be seen also as a rather focal damage (Harris et al., 2016). Further, in a large survey of clinical studies on neurodegenerative diseases, the authors have come to the conclusion “that hyperconnectivity is a common response to neurological disruption” (Hillary et al., 2015). Finally, recent clinical analysis of a group of patients with mild impairment in the early phase after stroke showed a globally noted increase in connectivity strength (S. Blaschke, Neurology Department, University Cologne; private communication) supporting our present findings in the mouse after a mild cortical stroke. Hypersynchronicity was also reported for lacunar stroke patients with aphasia (Yang et al., 2017). It should, however, also be mentioned that some clinical investigations focusing on aphasic stroke patients have reported connectivity decrease (Zhu et al., 2014; Nair et al., 2015; Sreedharan et al., 2019). But unfortunately, no clinical score on stroke severity was performed in those studies, so that lesion severity cannot be related to the direction of connectivity changes.

### Monitoring the Graft Fate

Intracortical grafting of the stem cells is the standard route of application, both in experimental and clinical settings (Guzman et al., 2018). We have investigated the stabilizing effect of intracortical implantation of hNSCs on the stroke-induced functional hyperconnectivity. For this purpose, it was important to monitor the graft vitality during the whole 12 weeks observation period. As had been shown in earlier studies, the vitality of the stem cells may decrease substantially with time after implantation losing a substantial portion of the originally implanted vital cells (Tennstaedt et al., 2015b; Vogel et al., 2018). We therefore followed the vitality of our graft by BLI of the cells stably transduced to express the ATP dependent luciferase signal. Our results showed that our hNSCs kept their full vitality for the entire monitoring period of 12 weeks after intracortical implantation, both, in healthy and in ischemic animals, thus assuring the conservation of the prerequisite condition of a vital graft to expect a persistent effect on the functional networks (Green et al., 2019).

The immunohistochemical analysis allowed no detection of grafted cells differentiated into GFAP^+^ astrocytes or NeuN^+^ neurons. In contrast to our findings, Vogel et al. (2019) had described that the majority of grafted hNSCs had differentiated into the glial lineage by 3 months when implanted into healthy brains of nude mice while these authors found only very few neurons. This difference may be explained by the fact that the human stem cells were not the H9-derived NSCs used here but of a different origin and were implanted only into the healthy cortex of nude mice. The ischemic environment may also influence the differentiation direction. In our earlier study on the presently used H9-derived NSCs grafted into the healthy nude mouse brain, we had observed only a weak signal of the synapsin-dependent BLI reporter after 3 months (Tennstaedt et al., 2015a) demonstrating the slow neuronal differentiation. This also explains that we could not detect NeuN^+^ neurons at 12 weeks. However, in animals, having survived an additional 4 weeks after implantation, we could convincingly show with patch-clamp electrophysiology that the graft had substantially differentiated into mature neurons, which was not yet visible 4 weeks earlier. Furthermore, these electrophysiological results proved, that the graft was following the neuronal lineage differentiation all along the 12 weeks observation period in the animals with implantation after stroke.

### Graft Induced Functional Network Stabilization After Stroke

Within a week after grafting the hNSCs adjacent to the ischemic cortex, the brain responded with complete suppression of the hyperconnectivity found in the stroke animals without grafting (*untreated stroke group*). This counteraction of the functional connectivity strength led to a small undershoot at week 2 relative to the pre-stroke baseline, but at 6 weeks post-stroke, the connectivity strength had closely approximated the baseline level, and at 12 weeks, it was not distinguishable from the normal condition. Persistence of this connectivity-stabilizing effect over the whole 12 weeks must be assigned to the continuously stable, viable graft, as we had earlier shown that persistence of graft vitality is necessary to preserve the normal functional network, otherwise deranged by the ischemic lesion (Green et al., 2019).

This observation of holding the functional connectivity close to or at the level of the baseline after stem cell grafting is supported by earlier observations where large and severe stroke lesions had led to a decrease in functional connectivity strength while stem cell grafts held the connectivity strength at baseline (Green et al., 2018).

This strong stabilization of the functional network by the stem cell graft occurs already at an early observation point after stroke induction, at a time when the stem cells cannot have differentiated into neurons yet, as was demonstrated by electrophysiology at 16 weeks after stroke. Thus, the stabilizing effect must rather be explained as a paracrine effect, probably of cytokine and growth factor secretion from the stem cells acting directly on the endogenous neurons or by modulating the inflammatory cells, in particular microglia and macrophages, to act mostly in a protective, anti-inflammatory mode.

## Conclusion

Using the stroke model of distal MCA occlusion in a nude mouse model for a well-described small cortical ischemic lesion, we have reported for the first time functional connectivity changes of the sensorimotor networks in this model of mild ischemia limited to the cortex. Different from large, severe strokes including the ischemic striatum which has been widely reported to show a longlasting *hypo*connectivity, here, a functional *hyper*connectivity is described in response to the mild cortical lesion.

Grafting hNSCs adjacent to the cortical ischemic territory and assuring persistence of graft vitality over the whole 12 weeks observation period, a rapid and complete stabilization of the functional networks at the level of baseline was found. This stabilization is interpreted to be most likely due to a paracrine effect as it occurs from the beginning after implantation, weeks before the neuronal differentiation was characterized by electrophysiology.

These different functional connectivity patterns leading to *hypo*connectivity in large, severe strokes but to *hyper*connectivity in small, mild strokes may be of great interest for clinical stroke diagnosis and functional outcome prediction. Finally, the reported stem cell-induced functional network stabilization generates expectation for translation from preclinical results to future clinical therapeutic application of stem cell treatment after stroke.

## Data Availability Statement

All datasets generated for this study are included in the article/Supplementary Material.

## Ethics Statement

All animal experiments were conducted following the German Animal Welfare Act guidelines and approved by the local authorities from LANUV (Landesamt für Natur, Umwelt und Verbraucherschutz Nordrhein Westfalen). The animal permission was approved under license 84-02.04.2014.A370.

## Author Contributions

AM: timeline design, all surgeries, cell transplantations, MRI acquisitions, MRI analysis, statistical analysis, BLI acquisitions, BLI analysis and manuscript writing. CG and DW: data analysis and manuscript writing. SV: cell culture, cloning, and transduction. MD: scripts for MRI data analysis and visualization. SH: electrophysiology recordings. MS: immunohistochemistry. MR: stroke surgery. PK: electrophysiological recording and analysis. MH: study design, data analysis and manuscript writing. All authors read and approved the final manuscript.

## Conflict of Interest

The authors declare that the research was conducted in the absence of any commercial or financial relationships that could be construed as a potential conflict of interest.

## Supplementary Material

FIGURE S1 T2-weighted MRI at the level of the hippocampus for the *sham-treated stroke group* and the *cell treated healthy group*. Representative animals for both groups without stroke are shown. The image slice position at the level of the hippocampus was chosen equivalent to the stroke cases given in Figure 3, i.e., the second most caudal section in Figure 2. In the sham animal, no thinning of the cortex was observed at the site of the sham surgery. In the healthy animal with stem cells implanted into the right cortex, no apparent lesion development was detectable over time.

FIGURE S2 Functional connectivity matrices of healthy animals during three repetitive measurements. The stability of the experimental protocol and reproducibility of the functional connectivity strength was assessed by submitting 10 healthy animals to three resting-state fMRI scans at 10-day intervals. The color-coded z-score matrices show a very stable and intra-group reproducible situation with only marginal variations between the three separate experiments.

TABLE S1 T2-MRI determined lesion volumes.
