# Supplementary figures and images for: Human Neural Stem Cell Induced Functional Network Stabilization After Cortical Stroke: A Longitudinal Resting-State fMRI Study in Mice
Source: Front Cell Neurosci. 2020 Apr 7;14:86. doi: 10.3389/fncel.2020.00086 (PMC7155295; doi:10.3389/fncel.2020.00086)

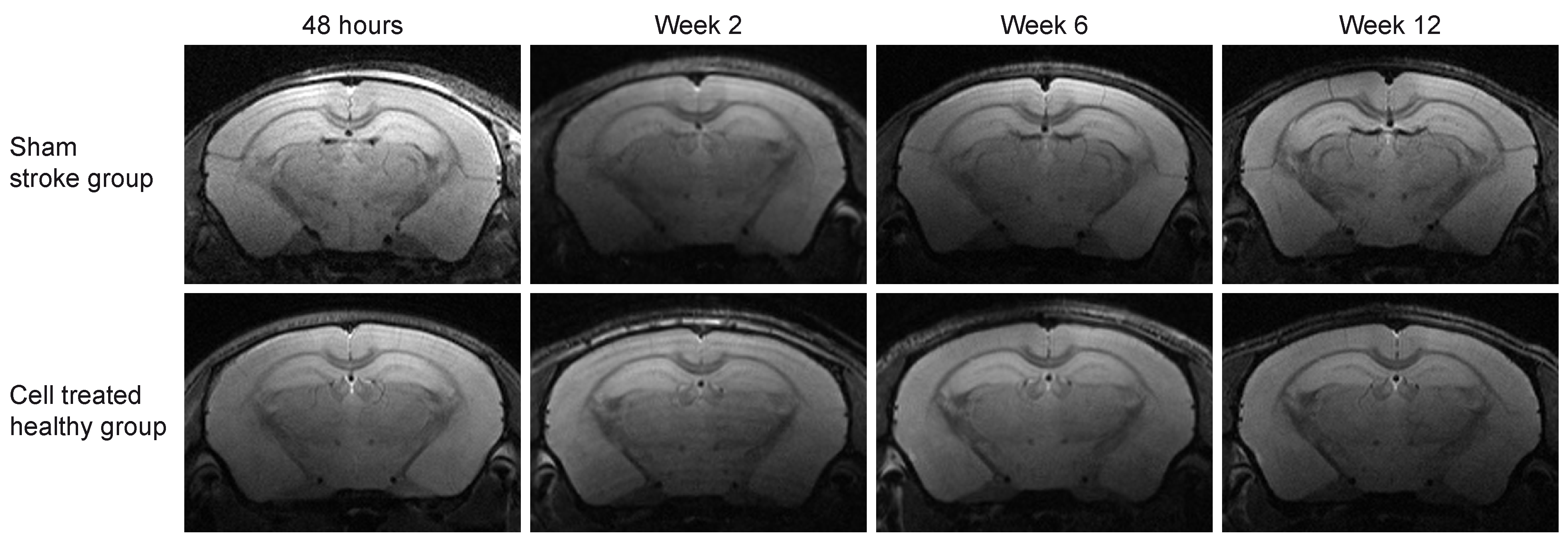

Supplement: FIGURE S1 — T2-weighted MRI at the level of the hippocampus for the sham-treated stroke group and the cell treated healthy group. Representative animals for both groups without stroke are shown. The image slice position at the level of the hippocampus was chosen equivalent to the stroke cases given in Figure 3, i.e., the second most caudal section in Figure 2. In the sham animal, no thinning of the cortex was observed at the site of the sham surgery. In the healthy animal with stem cells implanted into the right cortex, no apparent lesion development was detectable over time. [file Image_1.TIF]

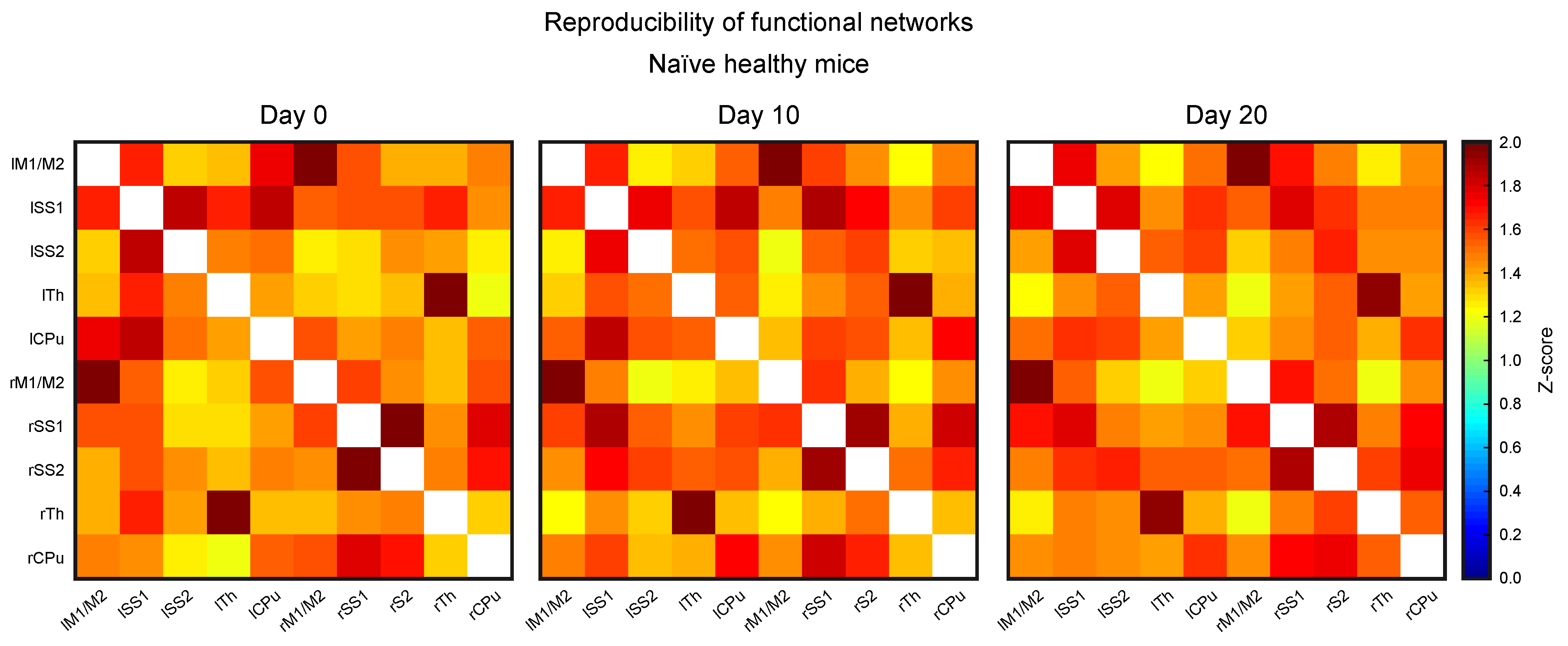

Supplement: FIGURE S2 — Functional connectivity matrices of healthy animals during three repetitive measurements. The stability of the experimental protocol and reproducibility of the functional connectivity strength was assessed by submitting 10 healthy animals to three resting-state fMRI scans at 10-day intervals. The color-coded z-score matrices show a very stable and intra-group reproducible situation with only marginal variations between the three separate experiments. [file Image_2.TIF]
