# Supplementary material for: Human Neural Stem Cell Induced Functional Network Stabilization After Cortical Stroke: A Longitudinal Resting-State fMRI Study in Mice
Source: Front Cell Neurosci. 2020 Apr 7;14:86. doi: 10.3389/fncel.2020.00086 (PMC7155295; doi:10.3389/fncel.2020.00086)
Supplement: TABLE S1 — T2-MRI determined lesion volumes. [file Table_1.docx]

**Suppl. Table 1**

**T2-MRI determined lesion volumes**

| Time after stroke induction | Groups | Lesion volume (mm^3^) | st dev |
| --- | --- | --- | --- |
| 48 hours | stroke untreated | 25.65 | 10.14 |
|  | stroke and cells | 32.12 | 8.22 |
|  |  |  |  |
| 2 weeks | stroke untreated | 5.68 | 2.15 |
|  | stroke and cells | 4.31 | 1.60 |
|  |  |  |  |
| 6 weeks | stroke untreated | 1.30 | 0.65 |
|  | stroke and cells | 1.38 | 0.67 |
|  |  |  |  |
| 12 weeks | stroke untreated | 0.43 | 0.33 |
|  | stroke and cells | 0.44 | 0.28 |
