# Supplementary material for: Human Neural Stem Cell Induced Functional Network Stabilization After Cortical Stroke: A Longitudinal Resting-State fMRI Study in Mice
Source: Front Cell Neurosci. 2020 Apr 7;14:86. doi: 10.3389/fncel.2020.00086 (PMC7155295; doi:10.3389/fncel.2020.00086)
Supplement: Supplementary file 4 [file Data_Sheet_1.PDF]

**Additional File 1:**

**Electrophysiology of whole cell patch clamp recording**

Electrophysiological experiments were performed on brain slices containing the cortex with engrafted hNSCs-eGFP 16 weeks after stroke induction and 15 weeks after grafting. For brain slice preparations, the mice were lightly anesthetized with isoflurane (B506; AbbVie Deutschland GmbH and Co KG, Ludwigshafen, Germany) and subsequently decapitated. The brain was rapidly removed and a block of tissue containing the cortex with the engrafted hNSCs-eGFP was immediately cut out. Coronal slices of 300 µm were cut with a vibrating microtome (HM-650 V; Thermo Scientific, Walldorf, Germany) under cold (4°C), carbogenated (95% $O_2$ and 5% $CO_2$), glycerol-based modified artificial cerebrospinal fluid (GaCSF; Ye et al., 2006) to enhance the viability of neurons. GaCSF contained (in mM) 250 glycerol, 2.5 KCl, 2 $MgCl_2$, 2 $CaCl_2$, 1.2 $NaH_2PO_4$, 10 HEPES, 21 $NaHCO_3$, 5 glucose, adjusted to pH 7.2 (with NaOH). Brain slices were transferred into carbogenated artificial cerebrospinal fluid (aCSF). First, they were kept for 20 min in a 35°C recovery bath and then stored at room temperature (24°C) for at least 30 min prior to recordings. The aCSF contained (in mM): 125 NaCl, 2.5 KCl, 2 $MgCl_2$, 2 $CaCl_2$, 1.2 $NaH_2PO_4$, 21 $NaHCO_3$, 10 HEPES and 5 glucose adjusted to pH 7.2 (with NaOH),.

Brain slices were transferred to a recording chamber (~3 ml volume) and continuously superfused with carbogenated aCSF at a flow rate of ~2 $ml*min^{-1}$. Experiments were performed at ~31°C using an inline solution heater (SH-27B, Warner Instruments, Hamden, CT, USA) operated by a temperature controller (Warner Instruments). hNSCs-eGFP were visualized with a fixed-stage upright microscope (BX51WI; Olympus, Hamburg. Germany), equipped with a 20x water immersion objective (XLUMplan FI; 20×; 0.95 numerical aperture; Olympus), a 4x magnification changer (U-TVAC, Olympus), and infrared-differential interference contrast (32) and fluorescence optics. In brain slices, engrafted hNSCs-eGFP were identified by their GFP fluorescence. Electrodes with tip resistances between 3 and 5 MOhm were fashioned from borosilicate glass (0.86 mm inner diameter; 1.5 mm outer diameter; GB150- 8P; Science Products, Hofheim, Germany) with a vertical pipette puller (PP-830; Narishige, London, UK). Patch pipettes were filled with intracellular solution containing (in mM): 141 K-gluconate, 10 KCl, 10 HEPES, 0.1 EGTA, 2 $MgCl_2$, 3 KATP, 0.3 NaGTP and adjusted to pH 7.3 (with KOH). For brain slice experiments the patch pipette was tip filled with internal solution and back filled with internal solution containing biocytin (1%; Sigma). Whole-cell current-clamp recordings were performed with an EPC10 amplifer

(HEKA, Lambrecht, Germany) and a modified ELC03-XS amplifier (NPI Electronic, Tamm, Germany) controlled by the software PatchMaster (version 2.32; HEKA). In parallel, data were sampled at intervals of 10 kHz with a CED 1401 using Spike2 (version 7) (both Cambridge Electronic Design, Cambridge, UK) and low-pass filtered at 2 kHz with a four-pole Bessel filter. The liquid junction potential between intracellular and extracellular solution was compensated (14.6 mV; calculated with Patcher's Power Tools plug-in (version 2.19) downloaded from http://www3.mpibpc.mpg.de/groups/neher/index.php? page1⁄4software for Igor Pro 6 [Wavemetrics, Lake Oswego, OR, USA]).To block voltage-gated sodium currents in brain slice recordings, tetrodotoxin (TTX; 1 µM; BN0517, Biotrend Chemikalien GmbH, Cologne, Germany) was added to the normal aCSF.

For single cell staining, brain slices were fixed in Roti-Histofix (P0873, Carl Roth, Karlsruhe, Germany) overnight at 4°C and subsequently rinsed in 0.1 M phosphate buffered saline (PBS; pH 7.2; three times for 15 min each time, RT). Brain slices were incubated for 2 days at 4°C in rabbit anti-GFP (1:1000; ab290; Abcam, Cambridge, UK) that was dissolved in PBS containing 1% Triton-X 100 and 10% normal goat serum. Afterwards, brain slices were rinsed in PBS (three times for 15 min each time) and incubated with the following secondary antibodies: dylight 650-conjugated streptavidin (1:200; ab134341; Abcam), dylight 488 anti-rabbit IgG (1:200; ab96883 ; Abcam) for 2h at RT in 1% Triton-X 100 in PBS. Brain slices were rinsed in PBS (5 times for 10 min each time), dehydrated, and then cleared and mounted in Permount (SP15B-500; Fisher Scientific, Nepean, Ontario, Canada). The fluorescence images were captured with a confocal microscope (Leica SP8) equipped with a 20x objective (HC PL APO 20x/0.75 IMM CORR CS2). Dylight 488 and 650 were excited with diode lasers at 488 and 638 nm, respectively. Emission of dyes was collected through the spectral detector of the Leica SP8. Confocal images were overlaid in ImageJ (version 1.45s) and final figures were prepared in Affinity Designer (Ver. 1.61, Serif Ltd., Nottingham, UK). Data analyses were performed with Spike2 (Cambridge Electronics) and Igor Pro 6 (Wavemetrics).
